# Workplace sitting is associated with self-reported general health and back/neck pain: a cross-sectional analysis in 44,978 employees

**DOI:** 10.1186/s12889-021-10893-8

**Published:** 2021-05-06

**Authors:** Lena V. Kallings, Victoria Blom, Björn Ekblom, Tobias Holmlund, Jane Salier Eriksson, Gunnar Andersson, Peter Wallin, Elin Ekblom-Bak

**Affiliations:** 1 Department of Physical Activity and Health, The Swedish School of Sport and Health Sciences (GIH), Box 5626, SE-114 86 Stockholm, Sweden; 2 Department of Public Health and Caring Sciences, Family Medicine and Preventive Medicine, Uppsala University, Uppsala, Sweden; 3 HPI, Health Profile Institute, Stockholm, Sweden

**Keywords:** Sedentary behaviour, Breaks, Exercise, Public health, Health risk, Self-reported health, Working population

## Abstract

**Background:**

Total sitting time is associated with a higher risk for cardio metabolic disease and mortality, while breaks in prolonged sitting attenuate these effects. However, less is known about associations of different specific domains and breaks of sitting on general health, back/neck pain and if physical activity could influence these associations. The aim was to investigate how workplace sitting and frequency of breaking up workplace sitting is associated with self-reported general health and self-reported back/neck pain.

**Methods:**

44,978 participants (42% women) from the Swedish working population, who participated in a nationwide occupational health service screening 2014–2019, were included in this cross-sectional study. Self-reported sitting duration and frequency of breaks from sitting at work, general health, back/neck pain, exercise, leisure time sitting, diet, smoking, stress and body mass index were assessed. Occupation was classified as requiring higher education qualifications or not. Logistic regression modelling was used to assess the association between workplace sitting/frequency of breaks in workplace sitting and poor general health and back/neck pain, respectively.

**Results:**

Compared to sitting all the time at work, sitting ≤75% of the time showed significantly lower risks for poor general health (OR range 0.50–0.65), and sitting between 25 and 75% of the time showed significantly lower risks (OR 0.82–0.87) for often reported back/neck pain. For participants reporting sitting half of their working time or more, breaking up workplace sitting occasionally or more often showed significantly lower OR than seldom breaking up workplace sitting; OR ranged 0.40–0.50 for poor health and 0.74–0.81 for back/neck pain.

**Conclusions:**

Sitting almost all the time at work and not taking breaks is associated with an increased risk for self-reported poor general health and back/neck pain. People sitting almost all their time at work are recommended to take breaks from prolonged sitting, exercise regularly and decrease their leisure time sitting to reduce the risk for poor health.

**Supplementary Information:**

The online version contains supplementary material available at 10.1186/s12889-021-10893-8.

## Background

Studies indicate an increased level of total sedentary time in the population [1]. Physical activity at work has decreased during the last half-century, with an increase of sitting time [2, 3]. Sitting is the most common sedentary behaviour and is defined as a position in which one’s weight is supported by one’s buttocks rather than one’s feet, and in which one’s back is upright [4]. The definition for sedentary behaviour is any waking behaviour characterized by an energy expenditure ≤1.5 metabolic equivalents (METs), while in a sitting, reclining or lying posture [4].

Sedentary time adjusted for physical activity level, is associated with a higher risk for cardiovascular disease, cancer and diabetes as well as for mortality [5–11]. A meta-analysis has also shown a dose-response relationship between increasing total sitting time and increasing risk of disease and mortality [12]. Breaks in prolonged sitting or reducing total sedentary time can counteract some of the negative effects of the cardio metabolic risks of sedentary behaviour [13–16]. The relationship between sedentary behaviour and self-reported health has been shown to be inconsistent [17–19]. Inconsistency has also been seen between sitting and pain in back and/or neck. A systematic review found few significant associations except for sitting time at work and lower back pain [20]. However, most studies have investigated total sedentary time and less is known about the health effects of sitting in different settings, such as during work or leisure.

Self-reported general health, defined as perceived overall physical and mental health, is associated with all-cause mortality and morbidity [21, 22]. During the last decades poor self-reported general health has increased markedly in all age groups and education groups in the working population in Sweden, [23]. Studies indicate different relationships between domain specific sitting time (work, transport and leisure time) and self-reported quality of life [19] but to our knowledge, studies are lacking concerning domain specific sitting in the working population and its association with self-reported general health. As sitting time increases at work it is important to study the association between sitting at work and health outcomes.

The aim of this study was to investigate how workplace sitting and frequency of breaking up workplace sitting is associated with self-reported general health and self-reported back/neck pain in a large national sample of men and women in the Swedish working force.

## Methods

The study was a cross-sectional study in the working population in Sweden. Data was obtained from the Health Profile Assessment database, which contains health profile assessments (HPAs) carried out in occupational health services in Sweden for almost 40 years to promote health [24]. Questions regarding sitting at work and frequency of breaking up sitting at work were added in January 2014, to the standard HPA questionnaire. Up until November 2019, a total of 44,978 participants had answered the questions regarding sitting at work and frequency of breaking up sitting at work, self-reported general health and back/neck pain and had provided data for the covariates. The number of cases with full data during the study period determined the sample size. The study adhered to the Declaration of Helsinki and was approved by the ethics board at the Stockholm Ethics Review Board (Dnr 2015/1864–31/2 and 2016/9–32). Informed consent was provided by all participants prior to data collection.

### Health profile assessment

The HPA is an interdisciplinary method [24, 25] with data collection and a person-centred dialogue with an HPA coach. The data collection and database are managed by the HPI Health Profile Institute (Stockholm, Sweden), which is also responsible for standardization of the methods used and education of the HPA coaches since its inception. Data are collected through an extensive questionnaire, and measurements of anthropometrics, blood pressure and estimation of VO_2_max based on a submaximal cycle test. The self-reported measures have been evaluated within the Health Profile Institute database since 1976. Participation is voluntary, free of charge, and offered to all employees working for a company or organisation connected to occupational or other health services.

### Workplace sitting habits

Workplace sitting was self-reported through the statement: *I sit at work …* with the alternatives *Almost all of the time, 75% of the time, 50% of the time, 25% of the time* or *Almost none of the time*. Frequency of breaking up workplace sitting was self-reported through the statement: *I break up my workplace sitting every 30th minute by at least standing up …* with the alternatives *Never, Seldom, Occasionally, Often*, or *Very often*. As a relatively low number of participants answered *Never* (*n* = 579), *Never* and *Seldom* were merged into one alternative in the logistic regression analyses.

### Self-reported general health and back/neck pain

Self-reported general health was assessed through the statement: *I perceive my physical and mental health as*. *..* with the alternatives *Very poor, Poor, Neither good or bad, Good,* or.

*Very good*. Self-reported back/neck pain was obtained through the statement: *I have back/neck issues …* with the alternatives *Very often, Often, Sometimes, Rarely* or *Never*. Self-reported general health and back/neck pain were further dichotomized into *Very poor/poor* vs. *Neither good or bad, Good,* or *Very good*, and *Very often/Often* vs. *Sometimes, Rarely* or *Never*, and introduced as dependent variables in the logistic regression analyses.

### Covariates

Diet habits, tobacco smoking, stress at work, overall stress, leisure time sitting and exercise were all self-reported (Supplement 1). Body height was measured to the nearest 0.5 cm using a wall-mounted stadiometer, and body mass was assessed in light-weight clothing to the nearest 0.5 kg using a calibrated scale. Body mass index (BMI, kg·m^− 2^) was subsequently derived. The Swedish Standard Classification of Occupations (SSYK) is a system for classifying and structuring occupations into administrative registers or statistical surveys. Occupation was reported according to the SSYK96 until June 2014 and according to the SSYK 2012 after that. SSYK was divided in two groups. SSYK 1–3 includes occupations requiring higher education qualifications or equivalent, i.e. high-skilled white-collar occupations such as managers, professionals (e.g. nurses, teachers), technicians and associate professionals (e.g. dental hygienists, police). SSYK 4–9 includes those with lower education qualifications, i.e. white-collar-low skilled (e.g. clerical support workers, service and sales workers) and blue-collar occupations (e.g. craft and related trades workers, machine operators and assemblers, elementary occupations).

### Statistical analysis

Continuous data were presented as mean with SD. The ordinal data obtained through questionnaire responses were further dichotomized according to the definition in Table 1. Significant trends with decreasing levels of sitting at work were tested for, using Kruskal–Wallis ANOVA (continuous data) and chi-square (ordinal data as proportions). Further, logistic regression models were used to assess the association between workplace sitting/frequency of breaks in workplace sitting and poor general health and back/neck pain, respectively. The models were adjusted for age, sex and SSYK (model 1); plus diet, smoking, stress at work, stress overall and BMI (model 2); plus leisure time sitting and exercise (model 3).

**Table 1:** Characteristics of the study population (*N* = 44,978) in relation to sedentary habits at work

	I sit at work …					
	Almost all time	75% of the time	50% of the time	25% of the time	Almost no time	
	*n* = 7354	*n* = 13,611	*n* = 11,430	*n* = 10,296	*n* = 2287	p-trend
Women (%)	47%	44%	40%	40%	38%	0.001
Age (yrs)	40.4 (10.9)	42.0 (11.3)	43.0 (11.5)	42.5 (12.2)	42.1 (11.6)	0.001
BMI (kg·m-2)	25.8 (4.7)	26.0 (4.6)	26.4 (4.5)	26.6 (4.6)	26.5 (4.6)	0.001
SSYK (1 to 3)	72%	69%	60%	40%	21%	0.001
Regular exercise (≥1 time/week)	68%	70%	69%	65%	58%	0.001
Diet (Good/very good)	64%	68%	68%	66%	68%	0.001
Smoking (never)	87%	87%	86%	82%	78%	0.001
Breaking up sitting at work (Seldom/Never)	26%	13%	5%	4%	4%	0.001
Low SED in spare time (< 50% of the time)	42%	49%	54%	61%	64%	0.001
Perceived Health (Very poor/poor)	10.1%	6.8%	6.5%	7.0%	6.3%	0.001
Perceived Back/Neck pain (Very often/often)	22%	19%	19%	21%	22%	0.001
Perceived stress at work (Very often/often)	23%	20%	18%	18%	21%	0.001

All values in mean (SD) or %

SSYK 1 to 3 includes high-skilled white-collar occupations, i.e. managers and occupations requiring higher education qualifications or equivalent

Subsequently, the total sample was stratified into sub-groups (men/women), SSYK level (Occupation requiring/not requiring higher education qualifications), exercise habits (no weekly regular exercise/weekly regular exercise), and sitting habits during leisure time (high leisure time sitting ≥50% of the time/low leisure sitting time < 50% of the time). The ORs (95% CI) associated with decreasing levels of workplace sitting within and between the sub-groups for very poor/poor general health and perceiving back/neck pain were calculated by logistic regression models adjusted for age, sex, SSYK, diet, smoking, stress at work, stress overall, BMI, leisure time sitting, exercise (when not stratified for). To test for interaction between men and women, occupations requiring higher education qualifications or not, regular exercise habits or not, high and low leisure time sitting, for change in odds ratio per decrease in sitting at work level (almost all time, 25–75%, almost no time), an interaction term (sex/SSYK/exercise habits/leisure time sitting) was introduced in the regression analyses. Significant interaction(s) were defined as *p* < 0.05 for the interaction term. All analyses were performed using IBM SPSS (version 25; SPSS Inc., Chicago, IL).

## Results

A total of 44,978 participants (42% women) with a mean age of 42.1 years (from 18 to 75 years old) were included in the analyses. Characteristics of the population divided by the five different categories of sitting at work are presented in Table 1. In total 72% of participants reported sitting at least half of the working day.

The group sitting almost all the time at work, included the highest proportion of women, had occupations requiring higher education qualifications, were youngest and had lowest BMI. Meanwhile they reported less breaks in sitting time, most sitting in leisure time, highest degree of self-reported poor health, back/neck pain and stress, and poorer diet. The group sitting almost none of the time at work, differed from the other groups with the lowest proportion of women, occupations requiring lower education qualifications, a lower proportion of exercising and more smoking (Table 1).

### Sitting time at work and general health

Increasing levels of workplace sitting were associated with having poor or very poor self-reported general health (Table 2). Sitting 75% or less of the time at work was associated with significantly lower risk for poor health compared to sitting almost all the time (OR range 0.50–0.65). These associations remained significant after multi-adjustment including diet habits, smoking, stress at work, stress overall, BMI (OR range 0.57–0.70, model 2). Exercise and leisure sitting (model 3) only slightly modified the associations for poor perceived poor self-rated health (OR range 0.67–0.82).

**Table 2:** Odds ratio (95% CI) for having poor perceived health and often perceived back/neck pain, respectively, in relation to level of sitting at work (N = 44,978)

	I sit at work …				
	Almost all time	75% of the time	50% of the time	25% of the time	Almost no time
	n = 7354	n = 13,611	n = 11,430	n = 10,296	n = 2287
**Perceived poor or very poor health**					
Model 1	1 (ref)	0.65 (0.58–0.72)	0.60 (0.54–0.67)	0.60 (0.54–0.67)	0.50 (0.42–0.61)
Model 2	1 (ref)	0.70 (0.63–0.77)	0.68 (0.60–0.77)	0.69 (0.61–0.78)	0.57 (0.46–0.71)
Model 3	1 (ref)	0.76 (0.68–0.86)	0.78 (0.69–0.88)	0.82 (0.72–0.93)	0.67 (0.53–0.83)
**Perceived back/neck pain often or very often**					
Model 1	1 (ref)	0.85 (0.79–0.91)	0.82 (0.76–0.88)	0.87 (0.81–0.94)	0.92 (0.82–1.04)
Model 2	1 (ref)	0.88 (0.82–0.94)	0.87 (0.81–0.94)	0.93 (0.86–1.00)	0.98 (0.87–1.10)
Model 3	1 (ref)	0.89 (0.83–0.96)	0.89 (0.82–0.96)	0.95 (0.88–1.03)	1.00 (0.89–1.14)

Model 1 Adjusted for age, sex and SSYK (occupation group);

Model 2 + Diet habits, smoking, stress at work, stress overall, BMI;

Model 3 + Leisure time sitting, Exercise

**Fig. 1:**
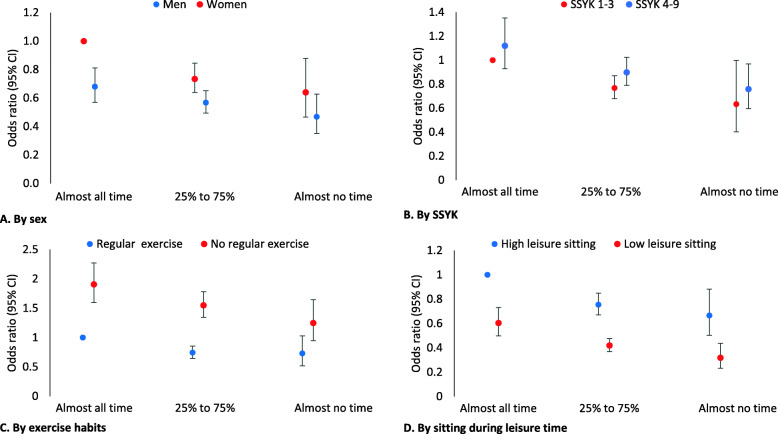
Odds ratio (95% CI) for perceived poor or very poor health with increasing levels of sitting at work in relation to **a**) sex, **b**) SSYK (occupation group), **c**) exercise habits, and **d**) sitting during leisure time. Adjusted (when not evaluated) for sex, age, diet, SSYK, smoking, stress at work, stress overall, BMI, leisure time sitting and exercise. There were no significant interactions between subgroups

Perceiving poor or very poor health with increasing levels of sitting at work in relation to sex, SSYK, exercise habits, and sitting during leisure time are presented in Fig. 1. Exact numbers can be found in supplementary file 2. There were no significant interactions between subgroups and sitting at work.

Women sitting almost all their time at work had the highest risk for poor health, while men had significantly lower OR 0.68 (0.57–0.81). Less sitting time at work was associated with a significantly lower risk for poor health in women (OR range 0.64–0.74), but not in men (Fig. 1a).

Occupations requiring higher education qualifications (SSYK 1–3) had a significantly lower OR 0.77 (0.68–0.87) for perceiving poor health, when sitting 25–75% of the time at work vs. sitting almost all the time (Fig. 1b). However, sitting at work did not significantly change the risk of poor health in occupations not requiring university competence (SSYK 4–9).

Exercise habits had a significant influence on the association between perceived poor health and sitting at work (Fig. 1c). When sitting almost all the time at work, the OR for poor perceived health was 1.91 (1.60–2.27) for no regular exercise compared to the reference regular exercise group. Exercising regularly was associated with significantly lower perceived poor health when sitting more than 25% of the working day. Within the regular exercise group, the OR for having perceived poor health was lower if sitting 25–75% of working time compared to sitting almost all the time.

Sitting during leisure time had a significant influence on the association between poor health and sitting at work (Fig. 1d). The low leisure time sitting group had a lower OR 0.60 (0.50–0.73) for poor self-reported health than the high leisure time sitting group when sitting almost all time at work. The risk difference for high or low leisure time sitting was found in all levels of sitting at work. In both the high and low leisure time sitting groups, a lower OR was found for not sitting all the time at work. The OR for perceived poor health for those with low sitting at both work and leisure time vs. high sitting at both work and leisure time was 0.32 (0.23–0.44).

### Sitting time at work and back/neck pain

Reporting back/neck pain often or very often was associated with increasing levels of workplace sitting. Sitting between 25 and 75% of the time at work showed significantly lower risk for back/neck pain compared to sitting all time (OR range 0.82–0.87). These associations remained significant for sitting 50 to 75% of the time after multi-adjustment for other lifestyle habits, exercise and leisure time sitting (OR was ≤0.89) (Table 2).

**Fig. 2:**
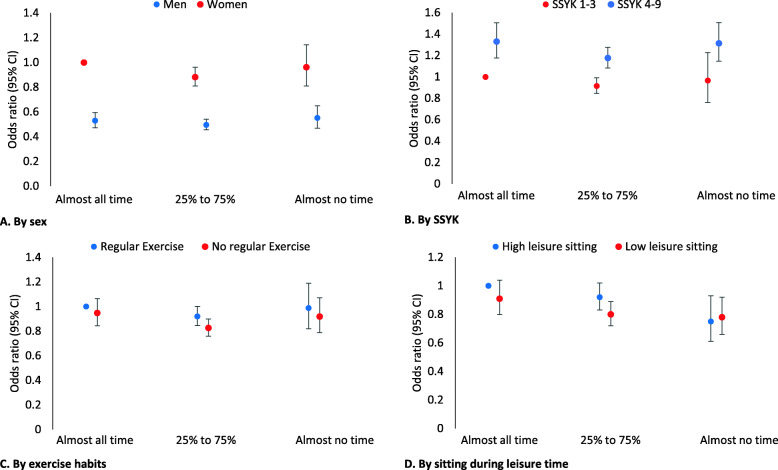
Odds ratio (95% CI) for perceived back/neck pain often or very often with increasing levels of sitting at work in relation to **a**) sex, **b**) SSYK (occupation group), **c**) exercise habits, and **d**) sitting during leisure time. Adjusted (when not evaluated) for sex, age, diet, SSYK, smoking, stress at work, stress overall, BMI, leisure time sitting and exercise. There were no significant interactions between subgroups

Perceiving back/neck pain often or very often with increasing levels of sitting at work in relation to sex, SSYK, exercise habits, and sitting during leisure time are presented in Fig. 2. Exact numbers can be found in supplementary file 3. There were no significant interactions between subgroups and sitting at work.

Sex significantly influenced the association between back/neck pain and sitting at work. Men had lower OR compared to women in all levels of sitting at work, and when sitting almost all time the OR was 0.53 (0.47–0.60). Women had significantly lower OR for perceiving back/neck pain when sitting 25–75% of the time at work vs. sitting almost all the time (Fig. 2a).

Occupations not requiring higher education qualifications (SSYK 4–9) had a significantly higher risk for perceiving back/neck pain than occupations requiring higher education qualifications (SSYK 1–3) when sitting 25% or more of the working time, with a OR of 1.33 (1.18–1.51) when sitting almost all the time (Fig. 2b).

Neither exercise habits nor leisure time sitting had any significant influence on the association between perceiving back/neck pain and sitting at work (Fig. 2c and d).

### Breaks in sitting at work and self-reported health

For participants sitting half or more (≥50%) of their working time, the association between frequency of breaking up workplace sitting every 30 min by at least standing up and poor or very poor self-reported general health and perceived back/neck pain often or very often respectively, are presented in Table 3.

**Table 3:** Odds ratio (95% CI) for having poor perceived health and often perceived back/neck pain, respectively, in relation to breaking up sitting at work in participants sitting 50% or more of the time at work (*n* = 32,395)

	Breaking up sitting at work every 30 min by at least standing up …			
	Seldom/Never	Occasionally	Often	Very often
	*n* = 4259	n = 10,872	*n* = 12,212	*n* = 5052
**Perceived poor or very poor health**				
Model 1	1 (ref)	0.50 (0.45–0.56)	0.44 (0.39–0.49)	0.40 (0.35–0.47)
Model 2	1 (ref)	0.62 (0.54–0.71)	0.59 (0.52–0.68)	0.55 (0.46–0.64)
Model 3	1 (ref)	0.67 (0.59–0.77)	0.69 (0.60–0.78)	0.65 (0.55–0.77)
**Perceived back/neck pain often or very often**				
Model 1	1 (ref)	0.81 (0.74–0.88)	0.74 (0.68–0.81)	0.76 (0.69–0.84)
Model 2	1 (ref)	0.89 (0.81–0.97)	0.83 (0.76–0.91)	0.87 (0.78–0.96)
Model 3	1 (ref)	0.90 (0.82–0.98)	0.85 (0.78–0.93)	0.90 (0.81–1.00)

Model 1 Adjusted for age, sex and SSYK (occupation group);

Model 2 + Diet habits, smoking, stress at work, stress overall, BMI;

Model 3 + Leisure time sitting, Exercise

An association was found between having poor or very poor self-reported general health and lower frequency of breaking up workplace sitting every 30 min. Breaking up workplace sitting occasionally or more often showed significantly lower OR than seldom breaking up (range 0.40–0.50). These associations remained significant albeit with a somewhat lower magnitude of OR after adjusting for other lifestyle habits, exercise and leisure time sitting (range 0.55–0.69) (Table 3).

An association was found between reporting back/neck pain often or very often and lower frequency of breaking up workplace sitting every 30 min. Breaking up workplace sitting occasionally or more often showed significantly lower OR than seldom/never breaking up (range 0.74–0.81). These associations remained significant albeit with a somewhat lower magnitude of OR after adjusting for other lifestyle habits (range 0.83–0.89), and after adjusting for exercise and leisure time sitting (range 0.85–0.90) (Table 3).

## Discussion

To our knowledge, this is the first study to analyse the associations between sitting at work and self-reported general health in a large sample of employed adults. The main results of the present paper are that both sitting almost all the time at work and not taking breaks in workplace sitting are associated with increased risks of self-reported poor general health and back/neck pain. Avoiding sitting almost all the time at work reduced the risk significantly for perceived poor health.

To our knowledge, there are no studies concerning the association between sitting time at work and general health, or on effects of breaks in sitting during work. Our results show an association between more sitting time at work and poor self-reported health. For people sitting at least half of their working time, breaking up workplace sitting occasionally or more often showed significantly lower risks for both poor health and pain, compared to those never taking breaks. This is in accordance with other studies on total sitting time or total sedentary behavior showing that high self-reported total sitting time relates negatively to health related quality of life [17, 19, 26, 27]. Moreover, domain specific sitting time was shown to be relevant in terms of back/neck pain. The group with high leisure time sitting had a lower risk for perceiving back/neck pain, when sitting almost no time at work vs. sitting almost all the time at work.

Breaking up sitting time was, in the present study, associated with a 10–15% decreased risk for perceived back/neck pain even after multi-adjustment (for age, sex and SSYK, diet habits, smoking, stress at work, stress overall, BMI, leisure time sitting, exercise). This is contrary to a systematic review where interventions to increase breaking up sitting among sedentary workers who had back pain did not decrease the back pain [28]. However, the interventions analysed in the review were mostly short-term (3–6 months) with small populations, so may not have been nuanced enough to pick up the decreased risk in back pain that was seen in our study. It would be interesting to evaluate if potential change in sitting- and breaking up sitting-time over many years could affect the outcome of back pain. Our results were partly in line with a study showing reduced pain in neck-shoulders, but not pain in the back, after an intervention reducing sitting time at work [29].

Several negative health effects of sedentary behaviour are more pronounced in physically inactive people [11]. High levels of exercise modify the risk for all-cause mortality in people with high sitting time [30]. However, others have found that prolonged sedentary behavior may weaken any protective effect of exercise on self-reported health [17]. The present study adds evidence that people who have to sit for long periods at work can decrease their risk by exercising. The present study showed that for workers sitting 25% or more of the working time, it is important to exercise regularly to reduce the risk of perceived poor health. For those sitting almost all the time at work, exercising regularly halved the risk for perceived poor health vs. not exercising regularly.

Earlier studies have shown mixed results of the effect of sex on the association between self-reported general health and total sedentary behaviour. These have shown either no significant differences between women and men [17], or that sex affects the relationship, however with the opposite results to this study in that it was the women who had a higher health-related quality of life than men [27]. In the present study, women showed higher risk compared to men for perceiving poor health when sitting almost all the time at work, and also for pain in the neck and back irrespective of sitting time at work. For men, sitting time at work did not significantly change the risk for perceived poor health or pain in back/neck.

In occupations requiring lower vs. higher education qualifications, the risk for perceived back/neck pain were higher when sitting 25% or more of the working time. Our results regarding sex and education differences are in accordance with an European study showing less musculoskeletal symptoms in men and those with higher education [31]. That men might have a lower risk for perceived back pain irrespective of time spent sitting compared to women can be because, generally, the prevalence of back pain for men is lower than women [32].

### Strengths and limitation

The strengths of this study are the large nationwide sample with over 44,000 working women and men, although the cohort may be somewhat selected as participation was not mandatory. Nor are all occupational health services included and no data exist on the number of subjects who were offered an HPA. The sample is unique in that all participants were employed at the time of assessment as well as including a great variation of occupations, so findings may be generalizable to larger populations of employed adults. Due to the large sample size, sub-group analyses were possible to conduct. The study includes four domain specific sedentary behaviour questions and relevant covariates for statistical adjustment. The main limitation of the study is the cross-sectional design exploring associations, which does not enable the direction of these relationships to be established. Data were based on self-report and non-validated questions. However, a similar question of total sitting time with the same five answer categories has been validated against accelerometer data and correlated (Spearman’s rho 0.5) with stationary time [33]. Self-reported general health, assessed with a similar question, has been validated and is in use in many population studies. It has for instance been shown to be a valid health status indicator in a Finnish working population [34]. If possible, more detailed information about work classification, rather than the crude work type classification used in this paper, should be used in future studies. Further studies are needed to confirm the results, such as randomized controlled trials to enable directions of relationships and a combination of different methods of assessing sitting and sedentary behaviour.

## Conclusions

Both sitting almost all the time at work and not taking breaks in workplace sitting are associated with an increased risk of self-reported poor general health and back/neck pain. To reduce the risk of poor self-reported health it seems important to reduce both total and prolonged sitting time at work as well as in leisure time. It is even more important to reduce sitting time at work for women and in those who do not exercise regularly to reduce the risk of self-reported poor health and/or neck and back pain.

People who have to sit almost all the time at work, should be recommended to take breaks from prolonged sitting, and to exercise on a regular weekly basis to reduce the risk of perceived poor health and neck and back pain. They should also decrease their leisure time sitting to reduce the risk of poor health.

## Supplementary Information

**Additional file 1.**

## Data Availability

The data underlying the findings in our study are not publicly available because the original approval by the regional ethic’s board (Stockholm Ethics Review Board, Dnr 2015/1864–31/2 and 2016/9–32) and the informed consent from the subjects participating in the studies did not include such a direct, free access. If a reader wants access to the data underlying the present article, please contact the HPI Health Profile Institute at support@hpihealth.se.

## Abbreviations

OR: Odds ratio
CI: Confidence interval
SD: Standard deviation
SSYK: Swedish Standard Classification of Occupation
