# Supplementary material for: Workplace sitting is associated with self-reported general health and back/neck pain: a cross-sectional analysis in 44,978 employees
Source: BMC Public Health. 2021 May 6;21:875. doi: 10.1186/s12889-021-10893-8 (PMC8101162; doi:10.1186/s12889-021-10893-8)
Supplement: Supplementary file 1 — Additional file 1. [file 12889_2021_10893_MOESM1_ESM.docx]

**Supplement 1. Questionnaire**

I consider my diet, regarding both meal frequency and nutritional content to be ...

*very poor, poor, neither good or bad, good, very good*

I smoke

*At least 20 cig/day, 11-19 cig/day, 1-10 cig/day, Occasionally, Never*

I perceive stress at work ...

*Very often, Often, Sometimes, Rarely, Never*

I perceive stress in my life, both personally and at work ...

*Very often, Often, Sometimes, Rarely, Never*

I sit at leisure time…

*Almost all of the time, 75% of the time, 50% of the time, 25% of the time, Almost none of the time*

I exercise for the purpose of maintaining/ improving my physical fitness, health and well-being ...

*Never, Sometimes, 1-2 times/week, 3-5 times/week, At least 6 times/week*

**Supplement 2.**

Odds ratio (95% CI) for perceiving poor or very poor health with increasing levels of sitting at work in relation to sex, SSYK, exercise habits, and sitting during leisure time.

|  | **I am sitting at work…** | |  |
| --- | --- | --- | --- |
|  | **Almost all time** | **25-75% of the time** | **Almost no time** |
| **By sex** |  |  |  |
| Women | 1 (ref) | 0.74 (0.64-0.85) | 0.64 (0.47-0.88) |
| Men | 0.68 (0.57-0.81) | 0.57 (0.49-0.65) | 0.47 (0.35-0.63) |
| **By SSYK** |  |  |  |
| SSYK 1-3 | 1 (ref) | 0.77 (0.68-0.87) | 0.63 (0.40-1.00) |
| SSYK 4-9 | 1.12 (0.93-1.35) | 0.90 (0.79-1.02) | 0.76 (0.60-0.97) |
| **By exercise habits** | |  |  |
| Regular Exercise >1t/week | 1 (ref) | 0.74 (0.64-0.86) | 0.73 (0.52-1.03) |
| No regular Exercise | 1.91 (1.60-2.27) | 1.55 (1.34-1.78) | 1.25 (0.95-1.65) |
| **By leisure time sitting** | |  |  |
| High leisure sitting | 1 (ref) | 0.76 (0.67-0.85) | 0.67 (0.50-0.88) |
| Low leisure sitting | 0.60 (0.50-0.73) | 0.42 (0.37-0.48) | 0.32 (0.23-0.44) |

**Supplement 3.**

Odds ratio (95% CI) for perceiving back/neck pain often or very often with increasing levels of sitting at work in relation to sex, SSYK, exercise habits, and sitting during leisure time

|  | **I am sitting at work…** | |  |
| --- | --- | --- | --- |
|  | **Almost all time** | **25-75% of the time** | **Almost no time** |
| **By sex** |  |  |  |
| Women | 1 (ref) | 0.88 (0.81-0.96) | 0.96 (0.81-1.14) |
| Men | 0.53 (0.47-0.60) | 0.50 (0.46-0.54) | 0.55 (0.47-0.65) |
| **By SSYK** |  |  |  |
| SSYK 1-3 | 1 (ref) | 0.92 (0.85-0.99) | 0.97 (0.76-1.23) |
| SSYK 4-9 | 1.33 (1.18-1.51) | 1.18 (1.08-1.28) | 1.31 (1.15-1.51) |
| **By exercise habits** | |  |  |
| Regular Exercise >1t/week | 1 (ref) | 0.92 (0.85-1.00) | 0.99 (0.82-1.19) |
| No regular Exercise | 0.95 (0.84-1.07) | 0.83 (0.76-0.90) | 0.92 (0.79-1.07) |
| **By leisure time sitting** | |  |  |
| High leisure sitting | 1 (ref) | 0.92 (0.83-1.02) | 0.75 (0.61-0.93) |
| Low leisure sitting | 0.91 (0.8-1.04) | 0.8 (0.72-0.89) | 0.78 (0.66-0.92) |
